# Deep Learning-Adjusted Monitoring of In-Hospital Mortality after Liver Transplantation

**DOI:** 10.3390/jcm13206046

**Published:** 2024-10-10

**Authors:** Nikolaus Börner, Markus B. Schoenberg, Benedikt Pöllmann, Philipp Pöschke, Christian Böhm, Dominik Koch, Moritz Drefs, Dionysios Koliogiannis, Joachim Andrassy, Jens Werner, Markus Otto Guba

**Affiliations:** 1 Department of General, Visceral, and Transplant Surgery, LMU, 81377 Munich, Germany; markus.schoenberg@gmail.com (M.B.S.); markus.guba@med.uni-muenchen.de (M.O.G.); 2 Transplantation Center Munich, LMU Munich, Campus Grosshadern, 81377 Munich, Germany; 3 Medical Centers Gollierplatz and Nymphenburg, 80339 Munich, Germany; 4 Institute of Informatics, LMU, 81377 Munich, Germany

**Keywords:** liver transplantation, surgery, risk adjustment

## Abstract

**Background**: Surgeries represent a mainstay of medical care globally. Patterns of complications are frequently recognized late and place a considerable burden on health care systems. The aim was to develop and test the first deep learning-adjusted CUSUM program (DL-CUSUM) to predict and monitor in-hospital mortality in real time after liver transplantation. **Methods**: Data from 1066 individuals with 66,092 preoperatively available data point variables from 2004 to 2019 were included. DL-CUSUM is an application to predict in-hospital mortality. The area under the curve for risk adjustment with Model of End-stage Liver Disease (D-MELD), Balance of Risk (BAR) score, and deep learning (DL), as well as the ARL (average run length) and control limit (CL) for an in-control process over 5 years, were calculated. **Results**: D-MELD AUC was 0.618, BAR AUC was 0.648 and DL model AUC was 0.857. CL with BAR adjustment was 2.3 with an ARL of 326.31. D-MELD reached an ARL of 303.29 with a CL of 2.4. DL prediction resulted in a CL of 1.8 to reach an ARL of 332.67. **Conclusions**: This work introduces the first use of an automated DL-CUSUM system to monitor postoperative in-hospital mortality after liver transplantation. It allows for the real-time risk-adjusted monitoring of process quality.

## 1. Introduction

Surgery in general represents a mainstay of medical care globally. Complications after surgical interventions play a major role in the recovery of patients. Liver transplantation specifically remains a high-risk surgical procedure despite constant improvements [1]. To ensure the safety of patients, it is important to prevent avoidable complications, which might be as high as over 50% [2,3]. Managing this risk includes structured preoperative planning, risk assessment, and early recognition, as well as immediate and appropriate management [4]. Structured preoperative planning as a prevention measure alone manages to reduce complications from 11.0% to 7% [5]. However, primary prevention alone cannot completely eradicate unnecessary complications. Currently, most methods for the analysis of remaining complications are based on manual analyses of quality indicators, combined with review procedures and external audits. These attempts to improve quality are carried out at intervals and are therefore retrospective [6]. In most cases, there is no risk adjustment but, at most, a risk grouping of patients. Thus, it is not always clear what the exact risk profile of patients with a complication was. Furthermore, retrospective analyses are problematic: when reacting too slowly, systemic quality issues can cost additional resources or, at worst, even patients’ lives [7].

We propose to solve this problem with a technique involving CUSUM charts [8,9]. Conventional CUSUM charts are known as a sequential analysis technique for process control within industry. A CUSUM chart shows the accumulation of events (mortality) in real time. It signals an out-of-control process by an upward drift of the cumulative sum graph until it crosses a predefined threshold. In medicine, this proven technique has not yet been broadly adopted. In the field of solid organ transplantation, prospective CUSUM charts are only performed in the OPTN/UNOS (Organ Procurement and Transplantation Network)/United Network for Organ Sharing) space for monitoring liver transplantations [10,11]. However, these analyses have no proper risk adjustment and therefore may lead to false-positive signals, especially in an increased proportion of high-risk patients. Conversely, this could lead centers to develop a bias towards transplanting low-risk patients, thereby passing over higher-risk individuals, who might be in greater need of the lifesaving organ. In the field of liver transplantation, a risk-adjusted CUSUM analysis might be particularly appropriate, as every donor or recipient presents with a distinctive set of risk factors. For this, however, accurate prediction is critical. In particular, considering the complexity of the association between donor and recipient factors, so-called deep learning (DL) neural networks for variable learning and selection might be particularly suitable [12]. Moreover, DL can continuously adapt and add new variables to the model through feedback (recursion) [13], which is ideal in an evolving and dynamic field such as transplantation.

In this paper, we present and test the first deep learning-adjusted CUSUM program (DL-CUSUM) to predict and monitor in-hospital mortality after liver transplantation. This study is unique as we developed and tested a novel DL algorithm to predict in-hospital mortality. Then, we combined this highly accurate prediction with a risk-adjusted CUSUM analysis to sequentially identify cases with excess mortality.

## 2. Materials and Methods

### 2.1. Study Groups and Predictive Variables

Patients receiving transplantation and their matching donors from 2004 to 2019 were included in the prospectively maintained database. Ethical approval was obtained from the institutional review board (EK 19-395, 08/2019) at the Ludwig-Maximilian University in Munich. The need for informed consent was waived by the institutional review board. This trial complies with the TRIPOD (Transparent reporting of a multivariable prediction model for individual prognosis or diagnosis) Statement.

All transplant patients were extensively evaluated, and other treatment options were considered. Clinical indication for transplantation was based on the recommendation of our multidisciplinary transplant board. During the wait time, all patients were regularly followed up and their indication was re-evaluated (either in an ambulatory or an in-hospital setting) [14].

In the case of an organ offer, experienced transplant surgeons evaluated the donor–recipient matching on a case-by-case basis. After acceptance, explanted organs were re-evaluated during cold preparation. Upon approval of the organ for transplantation, recipients were anesthetized and underwent hepatectomy. Standard liver transplantation in our institution is performed with the piggy-back technique but adapted accordingly. After transplantation, patients received a standard immunosuppressive protocol with Tacrolimus, MMF, and tapering dosages of steroids. In HCC patients, Tacrolimus is switched to Everolimus during follow-up visits if possible [15].

In this analysis, 62 preoperatively readily available variables from the recipient, the donor, and organ transportation data were included. The baseline recipient demographic variables were age, gender, diagnosis, weight, height, and blood type. The allocation variables included laboratory-measured Model of End-stage Liver Disease (MELD), allocation MELD, allocation modality, and high-urgency listing (HU). The recipient disease-specific variables were ascites, encephalopathy, and dialysis. Lastly, readily available laboratory values including Sodium (Na), Potassium (K), Creatinine (Crea), Albumin (Alb), Bilirubin (Bili), Aspartate Transferase (AST), Alanine Transferase (ALT), Gamma Glutamyl Transferase (GGT), Alkaline Phosphatase (AP), Hemoglobin (Hb), Leukocytes (WBC), Platelets (plt), C-reactive Protein (CRP), and the International Normalized Ratio (INR) were noted. Additional variables regarding the donor organ included the cold ischemia time (CIT), distance of procurement center, graft size, donor age, Donor Risk Index (DRI), cause of donor death, donor height, donor weight, donor BMI, donor gender, donor mechanical resuscitation, graft quality, donor Na, donor K, donor Crea, donor Alb, donor Bili, donor ASAT, donor ALAT, donor GGT, donor AP, donor Hb, donor Leuko and donor plt, donor INR, and donor CRP. These variables were chosen following an extensive systematic review of predictive variables for early mortality after liver transplantation [16]. From the above-mentioned variables, we calculated the compound scores for Body Mass Index (BMI), MELD, CTP (Child–Turcotte–Pugh Score), and DRI. These compound scores were not used for modeling since they heavily correlate with the variables from which they are calculated.

### 2.2. Follow-Up

According to international recommendations, transplant patients are structurally followed up. After an uneventful first year, the intervals are changed to every 6–9 months. Survival times for overall survival (OS) are calculated from the date of transplantation until the date of death. Because this study focuses on in-hospital mortality, no observations had to be censored.

### 2.3. Statistical Analysis

In general, normally distributed data were summarized with the mean and standard deviation (±SD) and compared using a *t*-test. Classification variables were compared using Fisher’s exact test. A *p*-value of <0.05 was considered statistically significant. All calculations were performed using the open-source software Python (Vers. 3.9.1, Python Software Foundation, Wilmington, DE, USA) RStudio (Version 1.1.463, RStudio Inc., Boston, MA, USA) and Prism Version 8.0 (GraphPad Software, Inc., La Jolla, CA, USA).

**Figure 1:**
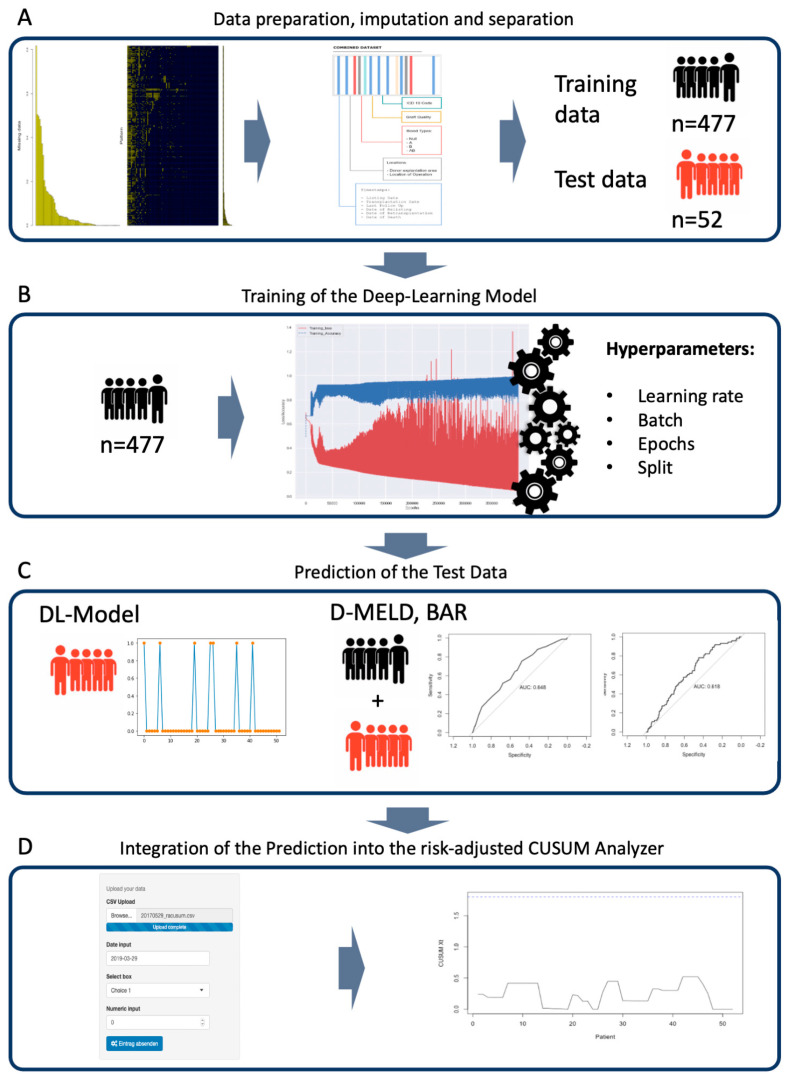
Workflow for the development and testing of the DL-CUSUM. A detailed description can be found in the Material and Methods section. BAR score, Balance of Risk; D-MELD, Donor age multiplied by recipient Model of End-stage Liver Disease.

Figure 1 depicts the steps of the development and validation of the DL-model, as well as the construction of the DL-CUSUM program, and Supplemental Figure S1 shows a graphical representation of the layers that are created during the modeling of the deep neural network for the DL-model (Figure 1A). First, preprocessing was carried out by imputing missing values with the novel MMCI Algorithm, which was specifically designed to calculate missing data in transplantation data sets [17]. In order to prevent biases, observations (donors or recipients) with more than 50% missing data and variables that could cause discrimination (anti-classification) were excluded from the analysis. After imputation, the cohort was split randomly 90% to 10% into a training and a test data set. This was in accordance with the TRIPOD statement [18].

The training data set was used to create the neural network and develop the DL-model. During development, the hyperparameters were tuned and cross-validated (CV). (Figure 1B). Hyperparameters that were tuned for the model included the learning rate, batch, epochs, and split between cross-validation groups. For further explanation, the rate is used to define how quickly the model is adapted to the problem. If the rate chosen is too small, the learning process takes too long; if it is too high, it might not adapt to the problem. In neural networks, batch and epochs are often confused. The batch defines the number of samples to train on before updating the model parameters. A training data set can contain more than one batch. In contrast, epochs are defined as the number of times that the algorithm will work through the training data set. Inherent to its nature, the DL model in this work is a so-called black box. That means that an analysis of the paths taken by the algorithm is not possible [19]. The test data were put aside and left untouched to be used for testing the DL-model after its development. The performance of the DL-model was evaluated using the area under the precision recall curve (PRAUC). For predictive machine learning models in imbalanced datasets, PRAUC is more informative than the area under the receiver operator curve (ROC) [20] (Figure 1C). Additionally, common and already-validated risk scores that incorporate recipient and donor data were used as a comparison for the novel DL-model. The D-MELD Score is calculated by multiplying the laboratory MELD score by the age of the donor [21]. The BAR (Balance of Risk) score comprises the recipient MELD, recipient age, donor age, cold ischemia time, whether recipients were on life support, and whether the recipient had received prior transplantations. A detailed description can be found in the original work by Dutkowski and colleagues [22]. The performance scores were calculated using the ROC.

To obtain a prediction of in-hospital survival, the scores were grouped according to published thresholds and retrospectively analyzed (Figure 1D). The predictions for in-hospital death ranged from 0 to 1 and were obtained from the DL-model, D-MELD, BAR, and mean in-hospital death. This individual death risk was integrated into a custom CUSUM Analyzer. With this, the risk-adjusted CUSUM analysis could be performed. These risk-adjusted CUSUM plots of expected vs. observed outcomes used the formula established by Steiner et al. [9]. The code of the CUSUM algorithm was obtained from the original publication from Steiner et al. [9]. The graphical user interface was constructed using the shiny app within the Rstudio software suite and made available online. Before drawing the CUSUM charts, the average run length (ARL) was calculated using the formula obtained by Steiner et al. [9]. The control limit (CL) was increased by 0.1 increments until the ARL was high enough to allow for continuous monitoring over 5 years without false-positive accumulations. The threshold of 5 years without false-positive accumulations was chosen according to the suggestions by Steiner et al. [9]. and the goal to balance between the ability for the program to run for a long time without false positives and the sensitivity provided by an accurate model. At about 60 transplantations per year, the threshold ARL was set at 300 to strike a balance between sensitivity and prevent signal fatigue. With this, the CL was adjusted to match the ARL. A lower CL indicated a more accurate prediction, since it minimized the chances for false-positive signals even if the control-limit is low. The ARL was tested through 1000 epochs (see definition above). With this, information in the CUSUM chart with the model-specific CL for the DL-model, D-MELD, BAR, and mean risk adjustment was drawn with patients from the test data.

## 3. Results

### 3.1. Patient Data

Five hundred and thirty-three patients received a liver transplantation during the period from 2004 to 2019. For these 533 matching donor observations were additionally added into the data base. Eight observations had to be excluded because of an excess of missing data. The demographic and clinical data for the transplanted patients are listed in Table 1.

Transplanted patients were 50.28 ± 12.29 years old. The average labMELD at transplantation was 23.79 ± 11.08. Because of the SE and NSE granted to qualifying patient allocation, MELD was 27.75 ± 8.55. Notably, albumin levels were decreased at 3.15 ± 0.67 g/L and transaminases were increased (ALT 328.94 ± 876.02 U/L, AST 454.85 ± 1318.16 U/L). The cholestasis parameter showed increased levels with bilirubin being 12.12 ± 13.56 mg/dL, GGT being 141.45 ± 186.77 U/L, and AP being 231.38 ± 252.37 U/L. Creatinine was increased at 1.66 ± 1.16 mg/dL. Also, INR was increased at 1.76 ± 0.90. After transplantation, patients stayed in hospital for 45.15 ± 39.87 days.

### 3.2. Transplantation and Donor Data

Accepted organs were 321.56 ± 210.99 km distant from the Transplantation Center in Munich. Consequently, the cold ischemia time was relatively high at 630.69 ± 156.61 min (Table 2).

Donors were 54.79 ± 16.27 years old. Overall, they had a calculated donor risk index of 1.98 ± 0.43. Albumin levels were decreased at 27.86 ± 6.46 g/L. Notably, when comparing the recipient data, inflammation parameters were increased with leukocytes at 13.85 ± 5.95 10^6^/L and CRP 14.78 ± 10.72 mg/dL. All donor data are listed in Supplemental Table S1.

### 3.3. Separation of the Data Sets and Training of the Deep Learning Model

After imputation and before the training and cross-validation of the algorithm, the study cohort was split by date of transplantation 90/10. With 529 transplantations altogether in the study group, the training data set included n = 477 and the test data set included n = 52 transplantations. After separation, the test data set remained untouched throughout the analysis and was only used for testing the final model [17]. Variables were compared between the training and the test data sets. Regarding recipients, all demographic disease-specific variables showed no significant difference. In the comparison of the laboratory values, potassium levels were significantly different between data sets (Table 1). Transplantation data showed a shorter distance from procurement to transplantation (*p* = 0.0215) in the test data. In the comparison of the donor data, the DRI was higher in the training data set (*p* = 0.0095). Training and hyperparameter calibrations were performed on the training data set. During this procedure, 600,000 epochs were calculated.

### 3.4. Predicting In-Hospital Mortality

As mentioned above, the test data set was used to measure the performance of the newly derived deep learning model. The DL model showed a strong predictive power with an area under the precision recall curve of 0.857. The AUROC of D-MELD of in-hospital mortality for the entire cohort (n = 529) reached 0.618. The BAR score reached an AUC of 0.648. Additional metrics are summarized in Supplemental Table S2.

### 3.5. Merging of Risk Adjustment and CUSUM Analysis

**Figure 2:**
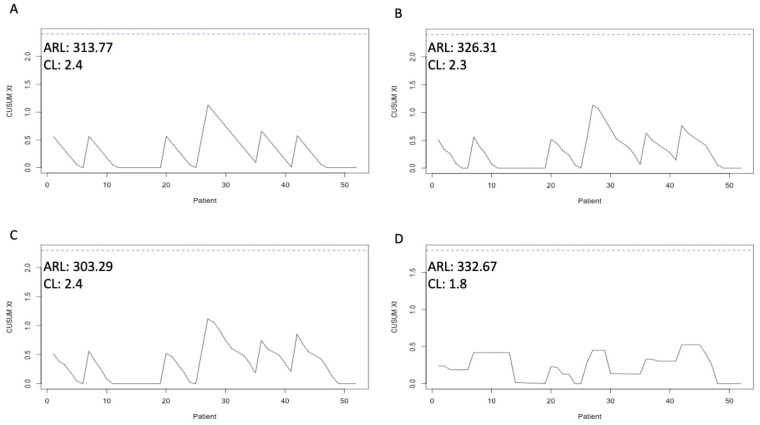
Risk-adjusted CUSUM (Cumulative Sum) charts with the corresponding CL (control limit) and ARL (average run length). (**A**) CUSUM chart for mean rate for in-hospital mortality, (**B**) CUSUM chart for BAR score (Balance of Risk), (**C**) CUSUM chart for D-MELD (Donor age multiplied by recipient Model of End-stage Liver Disease), (**D**) DL (deep learning-adjusted) CUSUM chart.

After predicting the in-hospital mortality of the patients in the test data set, we incorporated the prediction in the risk-adjusted CUSUM Analyzer. First, the mean rate for in-hospital mortality was calculated. At 13.80%, the CL Xt was set at 2.4 to reach an ARL of 313.77 (Figure 2A). When calculating the risk using BAR, the CL had to be set at 2.3 for an ARL of 326.31 (Figure 2B). With D-MELD, an ARL of 303.29 was possible with a CL of 2.4 (Figure 2C). With the risk adjustment performed by the DL algorithm, the CL could be set at 1.8 to reach an ARL of 332.67 (Figure 2D). With these CLs, risk-adjusted CUSUM charts could be drawn. In Figure 2, the risk-adjusted CUSUM charts with the corresponding CL and ARL are depicted. With the more accurate risk adjustment, neither false nor real excess mortality within a tighter control limit could be identified in the test data set. We have published the graphical user interface of the CUSUM Analyzer online. It can be found here: https://translationalsurgery.shinyapps.io/CUSUMAnalyzer/ accessed on 2 February 2022. When opening the online app, the reader can find instructions how to use the Analyzer.

## 4. Discussion

This work represents the first attempt to use the power of deep learning prediction to augment risk-adjusted CUSUM charts to monitor in-hospital mortality after liver transplantation [23]. We call this pipeline of interconnected algorithms DL-CUSUM. Transplantation programs are uniquely challenging to monitor, since outcomes are difficult to predict, dependent on the donor, and influenced by a large team of physicians from different disciplines [24]. Often, quality control is carried out periodically through retrospective analyses and is complicated because it requires a review of all cases [25].

With the DL-CUSUM, we set out to solve these problems. It is important to emphasize that transplant datasets differ from other clinical datasets in that they consist of multiple independent datasets (e.g., recipient data, donor data, and location data). Most AI models suffer from an incomplete data set on which the models are build. Within our data, we had less than 15% missing values. Imputing with standard means or deleting all datasets that had missing data would have resulted in fewer observations or significant noise within the data set. Thus, for this particular situation, we developed a new imputation algorithm that allows record segmentation (see above). The MMCI algorithm has already been tested using a historical transplant data set with 5, 10, 20, and 30% missing data (simulated). The MMCI showed the highest precision (>89%) in predicting missing data compared with conventional algorithms (random forest, MICE, and K-Nearest Neighbor) [18]. With this more complete data set, we developed and tested a deep learning algorithm based on known variables for predicting in-hospital mortality. With better prediction, cases that truly resulted in a preventable complication/mortality can be reviewed. We showed that the DL-CUSUM chart is superior to general risk and static risk adjustment using the D-MELD and BAR score. In fact, as demonstrated in the results, a less accurate prediction leads to deflection caused by the events stacking up at the wrong time point. In this case, a review of the wrong case could lead to the wrong conclusion being drawn [25]. However, with an accurate prediction, the DL-CUSUM system gains the capacity to analyze a case more thoroughly, in order to understand the events leading to a negative outcome for the patient. Also, with a more accurate prediction, the CL Xt can be reduced to allow for a higher sensitivity without sacrificing the average run length of 5 years (no false-positive signals over this time period). Some authors in the literature have suggested that an ARL of up to 30 years could be appropriate. With this, however, many accumulations would falsely be declared negative. Up to now, only CUSUM charts without modern risk adjustments have been used to monitor transplant programs [23]. With a preset risk, CUSUM charts can lead to biases and the investigation of an accumulation of cases that are falsely signaled as excess in-hospital mortality. To avoid signals that are too frequent, CL needs to be increased, which leads to a significantly decreased sensitivity in the monitoring. Also, a preset risk creates incentives for program physicians to treat patients with low risk for a negative outcome [25]. With accurate deep learning prediction, these problems can be solved. DL can continuously adapt and add new variables to the model through recursion [26]. With this, a dynamic model can be generated that rapidly benefits from new translational biomarkers or changes in practice [25]. Among the most promising new translational markers for the clinical status of liver transplant patients are so-called “frailty markers” [27]. It has already been shown that a multi-marker approach can sufficiently predict the elusive frailty syndrome [28]. Thus, it is ideal for the objective assessment of liver transplant patients.

The principles of the presented program are transferable to many other operations or interventions. In particular, high-volume interventions would benefit from an automated monitoring program to maintain oversight over the continuous development of operational quality. By automating this process, several endpoints (different types of complications, mortality, or time to discharge) could be monitored. For introduction into clinical practice, however a rapid identification of the cases causing the accumulation should be implemented. For this, we propose a strong pseudonymization with a key that never leaves the data repository of the treating institution. This would allow the safe storing of patient data. Additionally, for clinical introduction, some variables, such as the operating surgeon, should be excluded to prevent administrative abuse. An accumulation of events does not indicate a causal connection and should not be used for sanctioning [9].

This study and the algorithms used have limitations. First, a larger sample size would be desirable. Further, the results are based on a data set from one large transplant center that has transplanted patients from three Bavarian university hospitals, which makes it difficult to apply this model to a general patient population. However, the purpose was to use a pipeline of interlocking methods to showcase the combination of machine learning and CUSUM. In this work, it was possible to create an algorithm that accurately predicted the 10% of the data set that was set aside as test data. However, the test data consisted only of 52 transplantations. As shown in the tables, the test and the training data sets significantly differed for several variables. That allows us to be more confident that this algorithm, built on more than 450 transplantations, could be generalizable and not overly overfitted [17]. The use of a deep neural network might be overly complicated for this arguably relatively complete data set. However, the goal of this work was to create a workflow of intersected methodologies that could be used for any kind of data set and any kind of medical intervention. Deep neural networks can handle vast amounts of complex and difficult-to-interpret data and suit different outcomes where the effect of covariates may change over time. With the versatility of the workflow, this proof of concept can serve as a basis for further multicenter studies. The third limitation is natural for the algorithm used in this work. Deep learning algorithms are so-called black box algorithms. It is not possible to depict the decision process since the algorithm has so many layers. That means that after data are inputted, there is no possibility of a visualization or to check in any way how the model calculates the risk (Supplemental Figure S1) [19]. This is especially troubling if the input variables include gender or ethnicity. Since, for example, female individuals might have worse transplant outcomes, an agnostic algorithm could decide to discriminate against gender when predicting outcomes [29]. With white box methods like logistic regression, this type of bias is also possible. However, as the name suggests, white box algorithms can easily be interpreted for underlying biases. So how is it possible to prevent this from occurring when employing black box algorithms? If the relevant variables are directly available, then one strategy could be to make the algorithm unaware of this variable (basically, deleting the variable). This is called anti-classification [29]. Anti-classification can lead to a decrease in accuracy, which, however, can be accepted to a certain degree to prevent discrimination. In this study, we have excluded all variables which posed a direct threat of discrimination. As we aimed to monitor all liver transplant patients, we did not perform a diagnosis-based analysis, which, in the case of HCC, might have altered the outcome. As we aimed to create transparency and interpretability within our deep learning algorithm, we used only readily available clinical parameters. We like to emphasize that this study did not compare the practicality of the models, as traditional risk scores like D-MELD and BAR are well established and have proven valuable due to their simple utilization. However, within the experimental nature of this work, we hope to encourage further studies and research with deep learning-based prediction models, as they are sure to have a great impact in the future.

Another limitation of all result-driven monitoring concerns national and supranational data privacy laws. We concur with the fact that personal data should be secure and not available to the general public. The European Union has created a universal benchmark for data privacy. However, its interpretation and application vary from country to country. True real-time monitoring of anonymized/pseudonymized data could arguably have a more positive effect than potential data insecurities because even general trends can be used to learn from past mistakes [2,3].

## 5. Conclusions

This work introduces the first use of an automated DL-CUSUM system to predict and monitor post-transplant in-hospital mortality. Independent to our predictive model, we have created a novel CUSUM Analyzer with a graphical user interface that can be easily found online (https://translationalsurgery.shinyapps.io/CUSUMAnalyzer/ accessed on 2 February 2022). In future, similar systems could be used for any kind of intervention to allow for the real-time risk-adjusted monitoring of process quality.

## Figures and Tables

**Table 1:** Study data for the recipient study cohort. The training and test data are compared in terms of Body Mass Index (BMI), Model for End-stage Liver Disease (MELD), Alanine Transferase (ALT), Aspartate Transferase (AST), Gamma-Glutamyl Transferase (G-GT), Alkaline Phosphatase (AP), International Normalized Ratio (INR), and C-Reactive Protein (CRP; mg/L). SD, standard deviation.

Characteristics	Study Cohort	Training Data	Test Data	Training vs. Test
	n = 529	n = 477	n = 52	*p*-Value
**Demographics**				
Age at operation in years, mean ± SD	50.28 ± 12.29	50.06 ± 12.46	52.31 ± 10.58	0.2113
Male/female	357/172	318/159	39/13	0.2755
Height (m), mean ± SD	1.73 ± 0.10	1.73 ± 0.10	1.73 ± 0.09	0.9754
Weight (kg), mean ± SD	77.57 ± 16.39	77.79 ± 16.36	75.58 ± 16.66	0.3543
BMI, mean ± SD	25.67 ± 4.59	25.74 ± 4.57	25.03 ± 4.44	0.2903
**Liver disease features**				
Ascites, Y/N	332/197	301/176	31/21	0.6518
Encephalopathy, Y/N	216/313	194/283	22/30	0.8822
Dialysis, Y/N	77/452	72/407	5/47	0.2921
MELD, mean ± SD	23.79 ± 11.08	23.86 ± 11.16	23.17 ± 10.50	0.6710
Allocation MELD, mean ± SD	27.75 ± 8.55	27.83 ± 8.66	27.15 ± 7.64	0.5912
**Laboratory values**				
Na mmol/L, mean ± SD	135.98 ± 5.42	135.98 ± 5.43	135.98 ± 5.38	0.9983
K mmol/L, mean ± SD	4.10 ± 0.50	4.11 ± 0.49	3.95 ± 0.55	0.0268
Bilirubin mg/dL, mean ± SD	12.12 ± 13.56	12.02 ± 13.32	12.97 ± 15.83	0.6296
Albumin g/L, mean ± SD	3.15 ± 0.67	3.15 ± 0.68	3.16 ± 0.60	0.8627
ALT U/L, mean ± SD	328.94 ± 876.02	306 ± 829.33	421.81 ± 1023.03	0.0967
AST U/L, mean ± SD	454.85 ± 1318.16	389.63 ± 1125.18	684.92 ± 1854.65	0.3536
GGT U/L, mean ± SD	141.45 ± 186.77	140.23 ± 186.29	144.37 ± 189.98	0.8796
AP U/L, mean ± SD	231.38 ± 252.37	225.67 ± 251.54	246.48 ± 237.75	0.5693
Hemoglobin g/dL, mean ± SD	10.58 ± 2.50	10.60 ± 2.50	10.43 ± 2.47	0.6348
INR, mean ± SD	1.76 ± 0.90	1.77 ± 0.94	1.62 ± 0.51	0.2541
Creatinine mg/dL, mean ± SD	1.66 ± 1.16	1.65 ± 1.14	1.83 ± 1.30	0.2843
CRP mg/dL, mean ± SD	2.51 ± 3.58	2.50 ± 3.64	2.60 ± 3.09	0.8481
Leukocytes 10^6^/L, mean ± SD	8.15 ± 6.47	8.22 ± 6.66	7.50 ± 4.37	0.4426
Platelets 10^6^/L, mean ± SD	100.27 ± 74.17	100.49 ± 75.54	98.17 ± 60.68	0.8305

**Table 2:** Study data for the transplantation. SD, standard deviation.

Characteristic	Study Cohort	Training Data	Test Data	Training vs. Test
	n = 529	n = 477	n = 52	*p*-Value
Cold Ischemia Time (min) ± SD	630.69 ± 156.61	634.28 ± 159.66	597.77 ± 121.49	0.1104
Full/Split Liver ± SD	499/30	447/30	52/0	0.0607
Distance from Explanation to Transplantation (km) ± SD	312.56 ± 210.99	328.52 ± 210.31	257.73 ± 208.38	0.0215
Duration of Stay (Days) ± SD	45.15 ± 39.87	44.79 ± 39.64	48.42 ± 42.13	0.5334

## Data Availability

Data will be made available upon reasonable request and can be acquired by approaching nikolaus.boerner@med.uni-muenchen.de.

## Supplementary Materials

The following supporting information can be downloaded at: https://www.mdpi.com/article/10.3390/jcm13206046/s1. Supplemental Table S1: Study data of the donor study cohort. Training and Test data is compared. Alanine Transferase (ALT), Aspartate Transferase (AST), Gamma-Glutamyl Transferase (G-GT), Alkaline Phosphatase (AP), International Normalized Ratio (INR), C-Reactive Protein (CRP; mg/l), Standard Deviation (SD). Supplemental Table S2: Evaluation metrics of the models used in this analysis. DL-model (Deep-learning model), BAR Score (Balance of Risk), D-MELD (Donor age multiplied by recipient Model of End-stage Liver Disease) Supplemental Figure S1: Simple schematic display of the Deep Neuronal Network utilized.
