# Supplementary material for: Deep Learning-Adjusted Monitoring of In-Hospital Mortality after Liver Transplantation
Source: J Clin Med. 2024 Oct 10;13(20):6046. doi: 10.3390/jcm13206046 (PMC11508396; doi:10.3390/jcm13206046)

Supplemental Table S1: Study data of the donor study cohort. Training and Test data is compared. Alanine Transferase (ALT), Aspartate Transferase (AST), Gamma-Glutamyl Transferase (G-GT), Alkaline Phosphatase (AP), International Normalized Ratio (INR), C-Reactive Protein (CRP; mg/l), Standard Deviation (SD).

**Characteristics**	**Study Cohort**	**Training Data**	**Test Data**	**Training vs. Test**
	**n=529**	**n=477**	**n=52**	**P-Value**
**Demographics**				
**Age at operation in years, mean ±SD**	54.79 ±16.27	54.68±16.21	55.71±16.87	0.6669
Male/Female	271/258	241/236	30/22	0.3814
Height (m), mean ±SD	1.72 ±0.09	1.72±0.09	1.73±0.08	0.6766
Weight (kg), mean ±SD	77.81 ±14.71	77.88±14.94	77.21±12.48	0.7545
donor reanimation	136 (25.71%)	122 (25.58%)	14 (26.92%)	0.2429
Donor Risk Index	1.98±0.43	1.98±0.44	1.82±0.37	0.0095
**Laboratory Values**				
Na mmol/l, mean ±SD	147.9 ±8.18	147.93±8.10	147.60±8.93	0.7803
K mmol/l, mean ±SD	4.2 ±0.56	4.21±0.57	4.10±0.50	0.1627
Bilirubin mg/dl, mean ±SD	0.69 ±0.4	0.69±0.4	0.66±0.43	0.6129
Albumin g/l, mean ±SD	27.86 ±6.46	27.97±6.44	26.82±6.61	0.2242
ALT U/l, mean ±SD	65.72 ±132.3	65.24±137.34	59.98±71.44	0.7855
AST U/l, mean ±SD	83.52 ±135.27	82.84±137.30	90.46±115.87	0.7002
GGT U/l, mean ±SD	83.12 ±123.16	85.01±128.15	65.86±57.50	0.2874
AP U/l, mean ±SD	87.83 ±55.3	86.92±54.14	96.20±64.98	0.2506
Hemoglobin g/dl, mean ±SD	10.59 ±2.3	10.58±2.31	10.72±2.20	0.6897
INR, mean ±SD	1.24 ±0.53	1.24±0.54	1.24±0.43	0.9953
Creatinine mg/dl, mean ±SD	1.14 ±0.87	1.15±0.88	1.12±0.73	0.8153
CRP mg/dl, mean ±SD	14.78 ±10.72	14.78±10.51	14.86±12.64	0.9597
Leukocytes 10^6/l, mean ±SD	13.85 ±5.95	13.84±5.56	13.91±8.79	0.9380
Platelets 10^6/l, mean ±SD	191.02 ±103.2	189.69±103.70	203.19±98.59	0.3708

Supplemental Table S2: Evaluation metrics of the models used in this analysis. DL-model (Deep-learning model), BAR Score (Balance of Risk), D-MELD (Donor age multiplied by recipient Model of End-stage Liver Disease)

	**Area under the ROC Curve**	**Area under the Precision Recall Curve**	**Root Mean Square Error**	**Relative Absolute Error Loss**
**DL-model**	1.00	0.857	0.200	0.445
**BAR Score**	0.618	0.091	0.339	0.981
**D-MELD**	0.648	0.164	0.342	0.980

Supplemental Figure S1: Simple schematic display of the Deep Neuronal Network utilized.

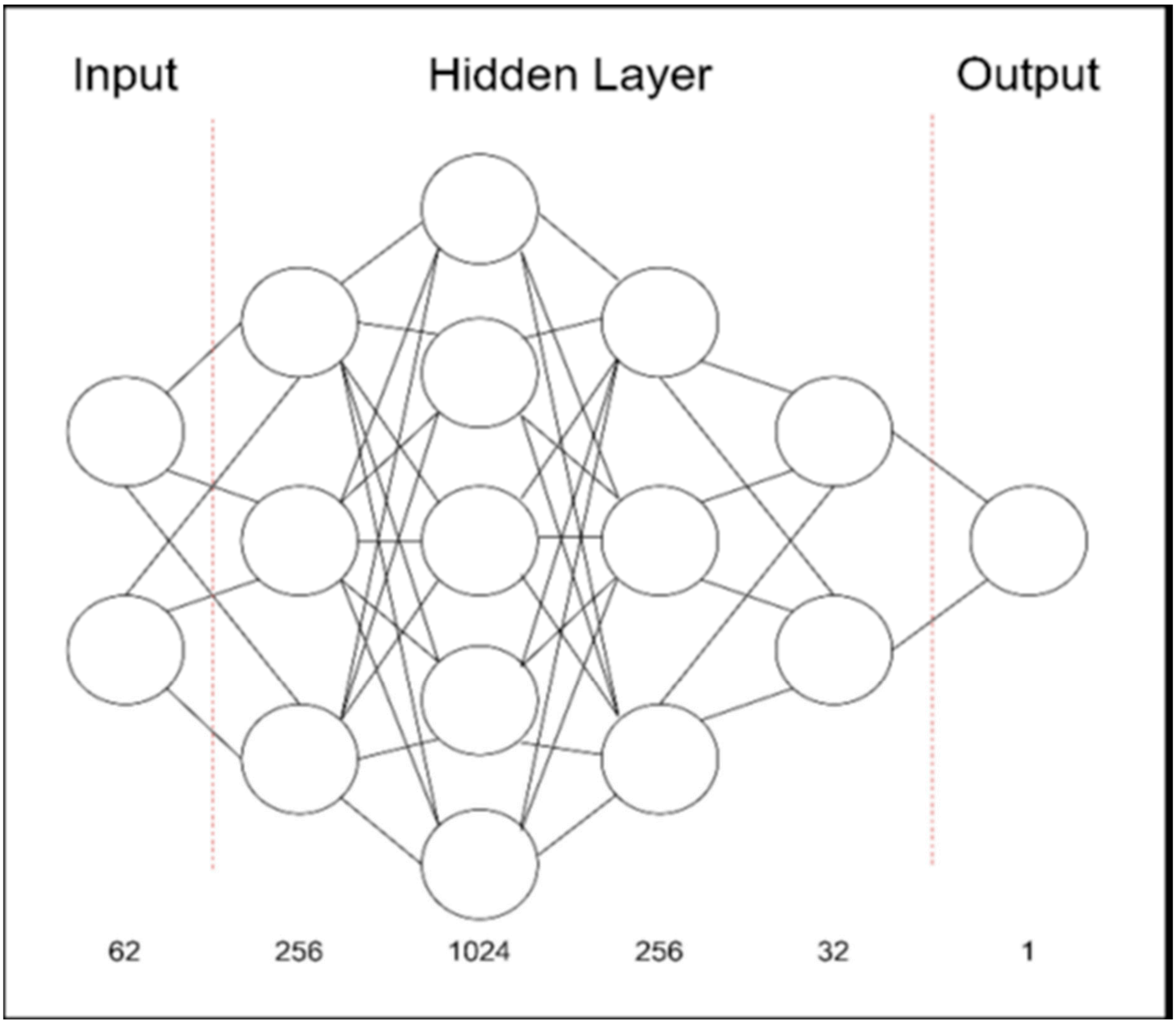

Supplement: Supplementary file 1 [file jcm-13-06046-s001.zip › jcm-3177309-supplementary.pdf]
